# Exposure to different light wavelengths impacts cortical areas associated with oculomotor and attentional control

**DOI:** 10.1371/journal.pone.0358046

**Published:** 2026-09-15

**Authors:** Cleofé Peña-Gómez, Bernat Sunyer-Grau, Marc Argilés

**Affiliations:** 1 Barcelona Supercomputing Center, Barcelona, Spain; 2 Departament d’Òptica i Optometria (DOO), Universitat Politècnica de Catalunya, BarcelonaTech (UPC), Campus de Terrassa, Barcelona, Spain; 3 Centre for Sensors, Instruments, and Systems Development (CD6), Universitat Politècnica de Catalunya - BarcelonaTech (UPC), Barcelona, Spain; The Ohio State University, UNITED STATES OF AMERICA

## Abstract

Light stimulation has been used in research to treat health-related problems. However, the impact of different wavelengths in cortical areas related to vision remains unknown. In this study, we aim to investigate the effects of light exposure to three different monochromatic wavelengths on brain areas involved in vergence and oculomotor control. Participants underwent resting-state functional magnetic resonance imaging before and after one-minute exposure to monochromatic light, on three separate days for three different wavelengths corresponding to blue, green, and red light. Fractional amplitude of low-frequency fluctuations and regional homogeneity were used to investigate the changes in the regional intensity of spontaneous fluctuations after each passive light stimulation, for cortical regions involving vergence and eye movements, but also for the whole brain. We observed that green light exposure compared with the red and blue light increases the intensity on: i) the intersection of the intraparietal sulcus with the postcentral sulcus (bilaterally), and ii) the intersection of the left middle frontal gyrus with the precentral sulci, measured with fractional amplitude of low-frequency fluctuations and regional homogeneity. This study shows the potential impact of light exposure on brain areas involved in attentional control tasks such as oculomotor control, especially when comparing the effects of green and blue light. Future research can further explore the impact of light stimulation in cortical regions involving the visual and oculomotor systems.

## Introduction

Light stimulation has therapeutic applications in cognitive neuroscience and medicine across a range of neuropsychiatric conditions [1]. In this context, light-based interventions have been shown to improve cognitive performance, alleviate headaches and migraines, ameliorate sleep disorders in adolescents, improve sleep disturbances in older adults with dementia, and reduce symptoms of seasonal affective disorder and nonseasonal depression [2–6]. In parallel, research in vision science has demonstrated that exposure to specific light wavelengths can enhance or modulate visual functions. For example, blue light stimulation has been shown not only to increase the speed of saccadic eye movements but also to improve spatial attention deficits associated with right hemisphere damage [7].

The neural bases of eye movements are well established [8]. Saccades rapidly shift both eyes in the same direction to redirect gaze toward a new location, whereas vergence movements rotate the eyes in opposite directions to maintain focus on objects at different depths. Distinct but partially overlapping neural networks, encompassing both subcortical and cortical regions, have been identified as supporting the control of saccadic and vergence movements [9].

Despite these functional distinctions, saccadic and vergence eye movements are tightly integrated during natural viewing, as gaze shifts across varying distances and directions typically engage both systems simultaneously. Using functional MRI (fMRI) Alkan et al. (2011), investigated and compared neural activity associated with saccades and vergence movements. Their findings revealed movement-specific activation patterns in the frontal eye fields (FEF) and midbrain, whereas activity in the supplementary eye fields, dorsolateral prefrontal cortex, cuneus, and precuneus was largely similar across both movement types, suggesting shared neural substrates supporting eye movement control [10].

Notably, the allocation of visual attention engages the dorsal attentional (DA) network, including parietal and frontal regions as well as the superior colliculus, with key contributions from the lateral intraparietal area and the FEF. Thus, many of the same regions involved in saccadic and vergence eye movements also play a central role in directing visual attention [11,12]. Furthermore, previous work has shown that brief passive light stimulation (1 min) using red, blue, and green monochromatic wavelengths can reorganize whole-brain functional connectivity (FC), particularly within attentional networks such as the salience and DA networks [13]. Taken together, these results suggest that cortical substrates involved in vergence, and oculomotor control also may be sensitive to external modulators of attention, such as light.

Therefore, building on this body of evidence, the present study focuses on cortical regions involved in vergence and oculomotor control, brain areas that are also implicated in attentional processing. This targeted approach aims to assess whether specific cortical regions are sensitive to wavelength-dependent modulation. To this end, we assessed wavelength-specific differences in regional brain activity following brief exposure to red, blue, and green light using the fractional amplitude of low-frequency fluctuations (fALFF) and regional homogeneity (ReHo). These measures are commonly used to characterize spontaneous neural activity in the resting state [14].

## Materials and methods

### Participants

Seven young adults, 4 females and 3 males, median age and standard deviation (±) 28.0  ±  4.50 years (range 21–33 years) were analyzed. All seven participants completed the three experimental sessions, however, the images from one subject in the blue condition were not used due to data corruption. The study followed the principles of the Declaration of Helsinki and was conducted under the approval of the Ethical Committee of the Hospital of Mutua de Terrassa, Spain (reference 07/2019). Informed written consent with the relevant information was given and accepted by all participants. The recruitment phase started on 01/10/2019 and ended on 02/11/2020.

Eligible participants were adults between 18 and 35 years of age who had not received light exposure therapy within the six months prior to study initiation. Exclusion criteria comprised neurological conditions, retinal diseases, color blindness, and refractive errors greater than 4 diopters of myopia or hyperopia or greater than 1 diopter of astigmatism. In accordance with fMRI safety guidelines, individuals with metallic implants or foreign bodies were excluded. Participants underwent a medical examination by a licensed medical doctor to ensure compliance with all study and scan requirements. Also, all participants were instructed to abstain from caffeine and alcohol and to avoid substantial changes in diet or sleep patterns during the 24 hours preceding the experiment. None of the seven participants were high caffeine consumers (over 300 mg/day), so caffeine abstinence did not represent significant withdrawal. While sleep quality was not formally assessed, all participants were asked to maintain routine sleep patterns and report any disruptions. No participant reported sleep anomalies before any session.

### Study design and light device

**Fig 1:**
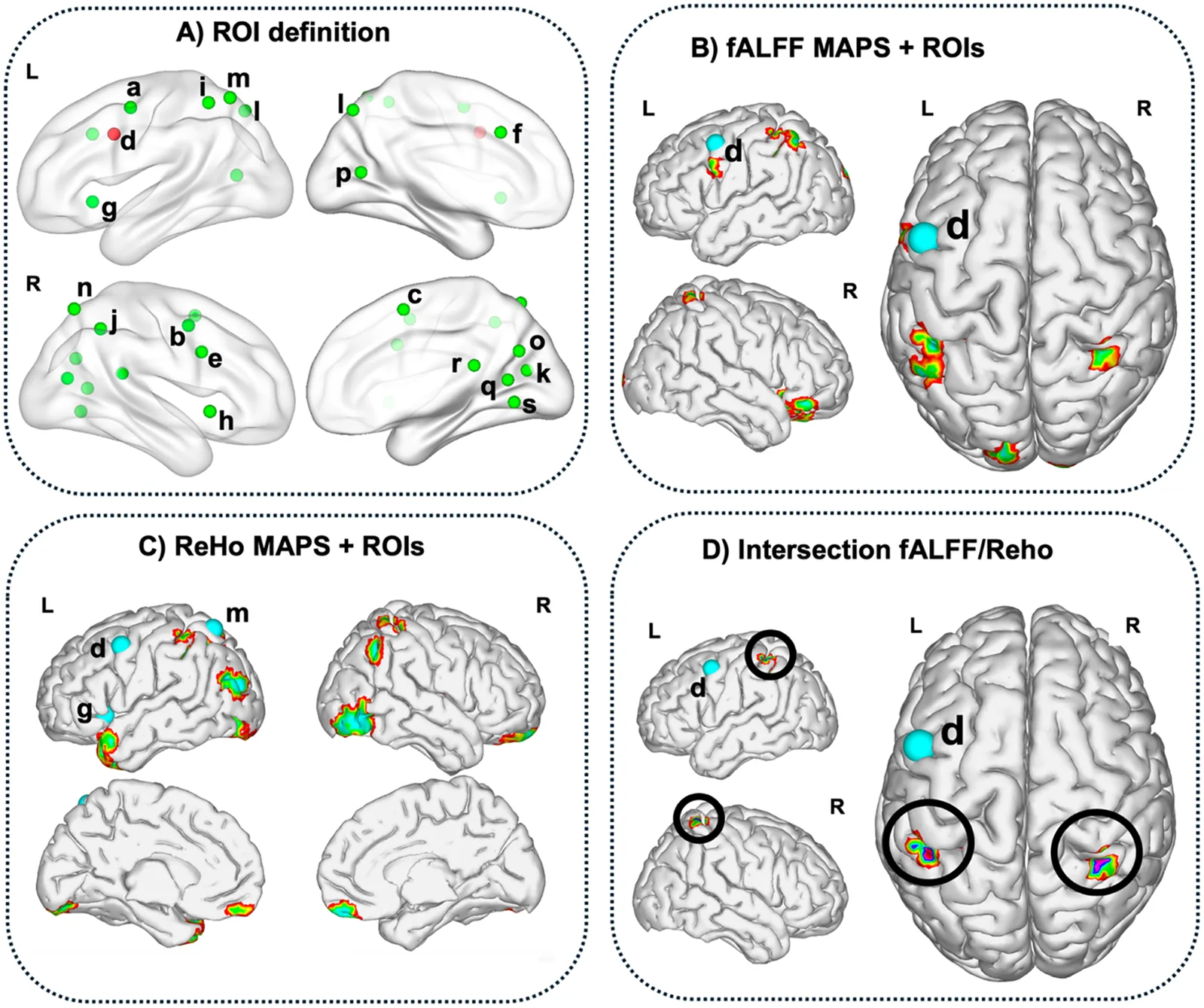
Results of the fALFF and ReHo analyses. (A) ROIs adapted from Alkan et al. (2011). (B) Significant fALFF increases after green versus blue/red light exposure across multiple parcellation granularities (300, 700, and 1000 regions), including seed d at the middle frontal–precentral gyri intersection. (C) ReHo results showing significant effects at seeds d (middle/prefrontal sulcus), g (insula), and m (superior parietal region). (D) Overlap between the fALFF and ReHo maps, highlighting bilateral parietal areas and seed d.

The experimental design consisted of a repeated measures design where all the subjects underwent a resting state functional magnetic resonance imaging (rs-fMRI) session before and after receiving one minute of light exposure to one of three wavelengths (green, blue, red). Each participant completed three rs-fMRI sessions on separate days, with a minimum interval of one week between sessions to minimize potential carryover effects across light conditions. All three sessions were scheduled on Saturday mornings at approximately the same time of day for each participant. This ensured that participants experienced comparable ambient light conditions when traveling to the lab, both between sessions and across participants. All participants were exposed to red, green, and blue light on the first, second, and third experimental days, respectively. Thus, the order of light exposure was fixed across participants. We also performed an additional analysis to control for intra-individual variability, using the intersection maps shown in Fig 1 and the ROIs listed in S3 Table. Imaging was performed using a 3-Tesla Philips MRI system (Ingenia CX, Philips Healthcare).

Light stimulation was delivered using a commercially available LED-based device, SIDTAV (Model FGM-02 (SIDTAV, Carrer Velázquez, 76, 08302, Mataró, Spain, https://sidtav.com/) designed for monochromatic light exposure. Three monochromatic conditions were tested: blue light (peak wavelength = 461.7 nm), green light (peak wavelength = 515.0 nm), and red light (peak wavelength = 633.7 nm). The spectral full width at half maximum (FWHM) was 22.3 nm for blue light, 37.4 nm for green light, and 18.5 nm for red light. Photopic illuminance at the eye was 0.86 lx, 21.99 lx, and 15.83 lx for the blue, green, and red conditions, respectively. Based on the CIE S 026 melanopic action spectrum, the corresponding melanopic equivalent daylight illuminance (melanopic EDI) values were 8.0 lx, 26.3 lx, and 0.072 lx, respectively. Photon densities for blue, green, and red light were 1.18 × 10¹⁹, 7.24 × 10¹⁹, and 1.68 × 10^20^ photons·m^−2^·s^−1^, respectively, with corresponding total irradiance values of 5.01, 27.7, and 52.7 W·m^−2^.

Participants arrived at the laboratory one hour before the first fMRI scan. During this period, they remained in an adjacent room, where they prepared for the scan and adapted to dim-light conditions. Transfer to the scanner room was also performed under dim-light conditions. Each fMRI scan lasted approximately 10 minutes. During image acquisition, participants’ eyes were completely covered with a sleep mask to prevent ocular exposure to ambient light (which was also dim). After completion of the first fMRI scan, participants were transferred to a second adjacent room, where the light exposure was administered. To minimize exposure to ambient light during the transfer, participants kept their eyes closed and were guided by a researcher. Once in the adjacent room, participants were instructed to place their head in the SIDTAV device and look directly at the light source for one minute. During this light exposure period, the room was kept in complete darkness. Immediately after light exposure, participants were transferred back to the scanner room, again with their eyes closed and guided by a researcher, for the second fMRI scan. The time interval between the end of light stimulation and the start of the second fMRI acquisition was approximately 5 minutes.

### Functional magnetic resonance imaging preprocessing

We used AFNI v20.1 for image processing, which included slice timing correction, realignment, normalization (MNI 152 EPI template; Montreal Neurological Institute, Montreal, Canada), and smoothing with a 6 mm FWHM Gaussian kernel. Also, we applied nuisance signal removal (CSF, white matter, movement), and scrubbing procedures to exclude extreme values from hardware instability or head motion. fALFF and ReHo calculations were performed using the DPABI toolbox [15].

### Fractional amplitude of low-frequency fluctuations analysis

fALFF was measured before and after light exposure to assess light-induced changes in the regional intensity of spontaneous brain activity. For fALFF computation, each voxel’s time series was transformed into the frequency domain via fast Fourier transform, yielding the power spectrum. For each voxel, the square root of the power spectrum was calculated at each frequency and averaged across the 0.01–0.08 Hz range. fALFF was then defined as the ratio of power within this low-frequency band to the power across the entire frequency range [16].

### Regional homogeneity

ReHo quantifies the similarity of the time series of a given voxel to those of its neighboring voxels, providing a voxel-wise measure of local functional synchronization. ReHo was computed using Kendall’s coefficient of concordance (KCC; [17]), yielding an individual standardized KCC map for each participant.

### Regions of interests

Nineteen regions of interest (ROIs) were defined as 6-mm-radius spheres based on coordinates reported by Alkan et al. (2011), who compared neural activation during fixation and random saccadic oculomotor tasks (see S1 Table for details). Each ROI was assigned a descriptive label, and the corresponding seed locations are illustrated in Fig 1A.

### Statistical analysis

For each ROI, we computed the change in fALFF between post- and pre-stimulation for each light condition. Statistical analysis was performed using a repeated-measures ANOVA with one within-subject factor (light exposure) for each ROI. In addition, whole-brain fALFF signals were extracted using Schaefer atlas parcellations at four spatial resolutions (100, 300, 700, and 1000 parcels; [18]). These varying parcellation scales were employed to examine the potential influence of ROI size on the detection of light-induced changes in fALFF intensity.

## Results

### fALFF on seeds and parcellations

The repeated-measures ANOVA conducted on the a priori–defined ROIs revealed a trend toward a main effect of light exposure for seed d (F = 3.54, p = .06), located at the dorsal junction of the middle frontal gyrus and the precentral gyrus (Fig 1A). Post hoc analyses indicated a significant increase in fALFF following green light exposure compared with blue light exposure (t = 3.55, p = .0045).

Whole-brain analyses using the 300-, 700-, and 1000-parcel Schaefer atlases identified several regions showing significant effects at an uncorrected statistical threshold (p < .01; Fig 1B), none of the *p*-values survived the false discovery rate correction. Peak effects were observed in the left supramarginal gyrus and intraparietal sulcus (IPS), the right superior parietal cortex, the inferior frontal gyrus (pars orbitalis), and visual cortical regions. Across these areas, fALFF values increased following green light exposure, particularly when compared with blue light exposure.

### ReHo on seeds and parcellations

The repeated-measures ANOVA revealed a significant main effect of light exposure in three a priori–defined ROIs: seed d, located at the dorsal junction of the middle frontal gyrus and precentral gyrus (F = 4.02, p = .04); seed g, corresponding to the insula (F = 7.92, p < .01); and seed m, located in the superior parietal lobule (F = 6.05, p = .01).

Whole-brain analyses of ReHo maps using the 300-, 700-, and 1000-parcel Schaefer atlases identified several regions showing significant effects at an uncorrected statistical threshold (p < .01), none of the p-values survived false discovery rate multiple comparisons correction in either the 19 seed-region tests or the analyses across the different parcellations used. Peak effects were observed bilaterally in the orbitofrontal cortex, as well as in the lateral visual cortex, the left IPS, the right superior parietal cortex, and the temporal pole (Fig 1C).

### fALFF combined ReHo

Fig 1D illustrates the overlap between fALFF and ReHo maps in the left IPS, the right superior parietal region, and seed *d*. A repeated-measures ANOVA on the mean values extracted from this intersection revealed significant effects of light exposure for both fALFF (F = 11.2, *p* < .01) and ReHo (F = 31.62, *p* < .0001). To assess the robustness of our results, we performed a leave-one-out analysis and found similar results (see S2 Table for details). For fALFF, the F values ranged from 7.39 to 14.10, and for ReHo, from 23.7 to 50.0; all iterations remained significant. For both measures, green light elicited the highest values, followed by red and then blue. Post hoc comparisons showed significant differences between green and blue (*t* = 4.7, *p* < .001) and between green and red (*t* = 4.8, *p* < .001), but no significant difference was observed between red and blue.

### Target mapping

**Fig 2:**
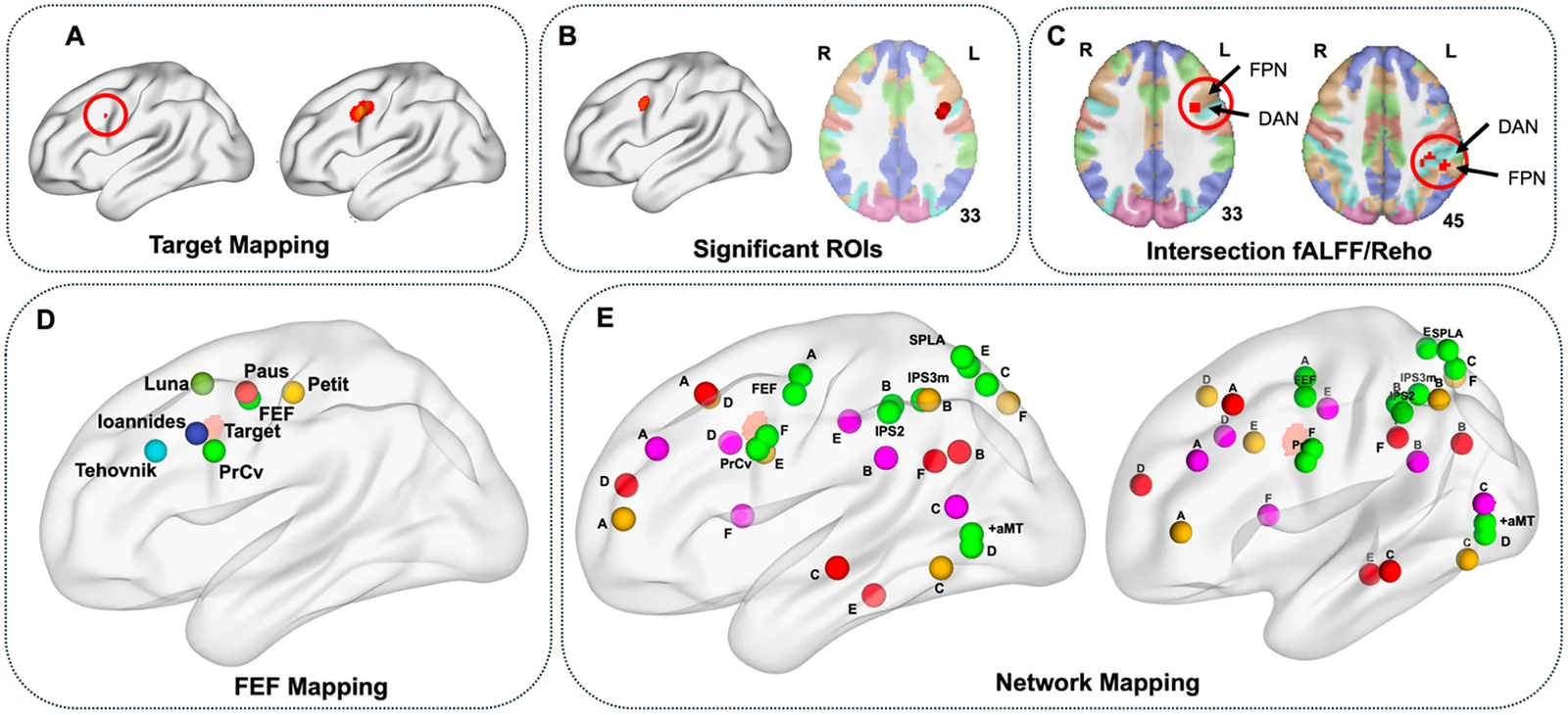
Mapping strategies. (A) Surrounding area mapping of seed *d* based on fALFF and ReHo results. (B) Significant seeds 1 and 2 (MNI −37, 0, 36 and −40, 5, 36) located between the DA and FP networks (center of gravity: −39, 3, 36 MNI). (C) Overlay of the fALFF–ReHo intersection on Yeo’s seven-network parcellation, positioned between the DA and FP networks. (D) FEF and the PrCv seeds derived from neuroimaging studies (see S4 Table). (E) Association cortex seeds for the DA (green), salience (magenta), FP (orange), and DM (red) networks (Yeo et al., 2011). In panels B and C, the numbers in the upper-right corner of each axial slice indicate the MNI coordinates.

Seed *d* exhibited a significant effect for ReHo and a trend-level effect for fALFF (p = .06). Accordingly, seven additional ROIs were defined around this region to assess fALFF effects (see S3 Table for MNI coordinates; Fig 2A; S1 Fig). Among these, seeds 1 and 2 (MNI coordinates: −37, 0, 36 and −40, 5, 36, respectively) showed significant effects of light exposure on fALFF (F = 4.4, *p* = .03; F = 5.5, *p* = .02), and also seed 1 for ReHo (F = 7.28, p = 0.009). Although none of the p-values survived false discovery rate correction for multiple comparisons in either modality. In both regions, fALFF values were lowest following blue light exposure and highest following green light exposure (Fig 2B).

### FEF mapping

The FEF are a key region of the DA network and have been shown to exhibit eye movement-specific patterns of activation. Therefore, we tested five seeds based on a review of the anatomy, localization, and function of the FEF by Vernet et al., 2014 [19] (see S4 Table), along with two additional seeds, the FEF and the ventral precentral region (PrCv), selected for their proximity to seed d and their well-established FC with the DA network (Yeo et al., 2011; Fig 2D). None of these regions exhibited significant effects of light exposure on fALFF.

### Network mapping

The regions showing overlap between fALFF and ReHo were located at the interface of the DA and frontoparietal (FP) networks (Fig 2C; see also S2 Fig for a detailed volumetric overlay on Yeo’s seven-network parcellation). Also, we analyzed 30 seeds distributed across the DA, FP, salience, and DM networks based on Yeo et al. (2011) (Fig 2E; see S5 Table for detailed coordinates).

Among these for the fALFF maps, only seed b in the DA network, located in the IPS (MNI −34, −38, 44), exhibited a significant effect of light exposure (F = 13.58, p = 0.001), this effect did survive FDR correction for multiple comparisons. The DA_F seed, located in the ventral precentral region (MNI −49, 3, 44), showed the second-highest F-value (F = 2.57, p = 0.12) but did not reach significance. For the ReHo maps, also the seed b located in the DA network (IPS, MNI −34, −38, 44) exhibited a significant effect of light exposure (F = 7.87, p = 0.07), however this effect did not survive FDR correction for multiple comparisons.

## Discussion

In this study, we examined wavelength-specific changes in neural activity using fALFF and ReHo, two measures that effectively capture spontaneous resting-state activity [14]. Green light exposure significantly increased both fALFF and ReHo values compared with red and blue light in the middle frontal gyrus–precentral sulcus intersection, the left IPS, and the right superior cortex, with blue light consistently producing the lowest values. These results suggest that different wavelengths can induce a dual effect on neural activity: modulating both the intensity of local signals (fALFF) and local synchrony (ReHo).

We observed increased fALFF in two significant seeds in the left premotor region (seed 1: MNI −37, 0, 36; seed 2: MNI −40, 5, 36) closer to the PrCv. A strong significant effect of light that survived multiple comparisons correction was also observed in the left IPS of the DA network (F = 13.5; Fig 2E), whereas FEF regions did not show significant effects. This pattern suggests that light effects are particularly pronounced in the IPS and may propagate to strongly connected regions such as the PrCv. These results align with previous research showing that FC between the IPS and PrCv is stronger than between the IPS and the FEF [20]. These findings are also consistent with prior work reporting modulation of attentional network connectivity, particularly enhanced frontoparietal connectivity following green light exposure [13]. Given that the DA network mediates goal-directed, sustained, and visuospatial attention [21,22], green light may prime cognitive control systems for goal-directed tasks, whereas red and blue light appear to have opposite or attenuated effects.

Through combined activation of melanopsin and cone pathways, green light may modulate retino–thalamo–cortical and retino–hypothalamic circuits [23]. Via projections to the lateral geniculate nucleus (LGN) and pulvinar, intrinsically photosensitive retinal ganglion cells (ipRGC) input may enhance sensory processing in occipital and parietal cortices, while projections to the hypothalamus and locus coeruleus (LC) can increase global cortical excitability [24]. These neurons exhibit peak spectral sensitivity around 480 nm and respond primarily to short-wavelength (blue) light, with partial sensitivity to green light [25]. Although ipRGCs are best known for their role in regulating circadian rhythms and the pupillary light reflex [26], accumulating evidence indicates that their projections beyond the primary visual system may also contribute to higher-order cognitive functions, including pattern and color vision [27,28].

Notably, LC activation is closely associated with arousal and attentional states, supporting its role in adaptive responses to salient environmental stimuli [29,30]. Noradrenergic projections from the LC partially converge on core regions of the DA network, which may account for the increases in fALFF and ReHo observed in the IPS and precentral frontal regions in the present study.

We hypothesize that this network-level modulation reflects a preparatory state that enhances cognitive control mechanisms relevant to visuospatial attention and oculomotor function. This interpretation should be tested in future studies incorporating task-based behavioral measures to directly assess the functional consequences of wavelength-specific light exposure.

Overall, the present study makes three key contributions. First, it moves from an exploratory whole-brain approaches to a more targeted examination of cortical regions implicated in oculomotor and attentional control. Second, by combining fALFF and ReHo metrics, the study captures complementary aspects of resting-state activity, thus, signal intensity and local synchrony, providing convergent evidence for wavelength-specific modulation of neural activity. Third, these findings provide a basis for future task-based and neuromodulation studies, in which green light exposure could be evaluated for its potential to enhance visuospatial attention, saccadic performance, or cognitive readiness.

Despite these findings, several limitations should be considered. First, the lack of behavioral measures prevents direct functional validation of the observed changes in brain activity. Future studies should therefore include behavioral paradigms such as saccadic reaction time, visual search, or vergence accuracy, administered before and after light exposure to assess functional outcomes more directly. Second, the relatively small sample size (n = 7) limits statistical power and generalizability, making the results more susceptible to individual variability. Third, the order of light exposure was fixed across participants and was not randomized or counterbalanced. Although the participants did not perform any cognitive task, and neither the researchers nor the participants had expectations or means to influence the results, potential order effects, habituation, fatigue, adaptation to the fMRI environment, or other session-related effects cannot be completely ruled out. To mitigate this, we performed an additional analysis to test intra-individual variability and found no differences (F = 3.5229, p = 0.22 for the intersection maps; F = 2.12, p = 0.12 (fALFF) and F = 1.05, p = 0.35 (ReHo) for the network mapping. Nevertheless, small sample sizes are common in fMRI pilot studies [31–33], particularly when within-subject designs are used, which partially mitigate these constraints. Even so, studies with larger cohorts will be necessary to confirm the robustness of the present findings and to further characterize inter-individual variability in responses to light exposure.

## Conclusions

This study represents a step toward linking sensory stimulation with large-scale cortical network modulation and its potential applications in both basic and translational neuroscience. Our findings indicate that brief light exposure, particularly the contrast between green and blue wavelengths, can modulate cortical regions involved in oculomotor control and attentional processing. These results provide preliminary evidence that may inform the design of future studies examining wavelength-specific effects of light in visual and cognitive neuroscience.

## Supporting information

S1 Table Coordinates for the fixation versus random saccadic oculomotor task in Talairach–Tournoux and MNI space, converted using the tal2icbm_spm.m function (Lancaster et al., 2007). Adapted from Table 1 in Alkan et al. (2011).
(DOCX)

S2 Table Leave-one-subject-out analysis for the fALFF and ReHo intersection maps. The original values for the fALFF, based on all subjects, were F = 11.02, p < 0.01, and fot the ReHo intersection maps, were F = 31.62, p < 0.0001.
(DOCX)

S3 Table Regions of interest defined to map the area surrounding seed d (MNI −34, 7, 36). Only seeds 1 and 2 showed significant effects of light on fALFF (*p* < 0.05).
(DOCX)

S4 Table MNI coordinates adapted from Vernet et al. (2011) and Yeo et al. (2011) for FEF mapping (see Figure 2D). (DOCX)

S5 Table MNI coordinates for network mapping adapted from Yeo et al. (2011; see Figure 2E). The set includes all 24 seeds reported in Table 5 of Yeo et al. (2011), along with six additional seeds, primarily from the dorsal attention network (PrCv, FEF, aMT, SPLA, IPS2, IPS3m), derived from Table 2 of the same study. *p = 0.001.
(DOCX)

S1 Fig ROIs surrounding the target (MNI, −34,7,36). (DOCX)

S2 Fig Intersection between fALFF and ReHo over Yeo’s Percellation. (DOCX)
