# Supplementary material for: Exposure to different light wavelengths impacts cortical areas associated with oculomotor and attentional control
Source: PLoS One. 2026 Sep 15;21(9):e0358046. doi: 10.1371/journal.pone.0358046 (PMC13577468; doi:10.1371/journal.pone.0358046)
Supplement: Supplementary file 1 [file pone.0358046.s001.docx]

**Table S1.** Coordinates for the fixation versus random saccadic oculomotor task in Talairach–Tournoux and MNI space, converted using the tal2icbm_spm.m function (Lancaster et al., 2007). Adapted from Table 1 in Alkan et al. (2011).

| **Regions of Interest** | **BA** | **Talairach-Tournoux** | **MNI** | **Label** |
| --- | --- | --- | --- | --- |
| Frontal Eye Field, Superior Middle Frontal Gyrus, Precentral Gyrus | 8/6 | -36 -9 50 | -37 -3 52 | a |
|  |  | 30 -5 48 | 34 1 48 | b |
| Supplementary Eye Field, Medial Frontal Gyrus | 6 | 1 -2 53 | 3 5 54 | c |
| Dorsolateral Prefrontal Cortex | 9 | -33 2 36 | -34 7 36 | d |
|  |  | 36 4 34 | 40 9 32 | e |
| Anterior Cingulate/ Cingulate Gyrus | 24/32 | -2 14 38 | -1 20 36 | f |
| Inferior Ventral Lateral Prefrontal Cortex, Inferior Frontal Gyrus, precentral Gyrus | 45/47 | -26 17 1 | -27 20 -5 | g |
|  |  | 41 11 2 | 45 14 -4 | h |
| Parietal Eye Field, Inferior Parietal Area | 40 | -33 -53 48 | -34 -50 55 | i |
|  |  | 29 -54 41 | 33 -52 46 | j |
| Cuneus, Lingual Gyrus | 17/18 | 8 -70 12 | 10 -72 16 | k |
| Precuneus | 7 | -5 -73 42 | -3 -72 50 | l |
| Superior Parietal Area | 7 | -30 -65 50 | -30 -63 58 | m |
|  |  | 26 -70 50 | 30 -68 58 | n |
| Posterior Cingulate | 31 | 1 -67 23 | 3 -67 28 | o |
|  | 30 | -7 -65 8 | -6 -67 11 | p |
|  |  | 6 -59 8 | 8 -60 10 | q |
|  | 29 | 0 -40 18 | 1 -39 19 | r |

**References**

1. Lancaster JL, Tordesillas-Gutiérrez D, Martinez M, Salinas F, Evans A, Zilles K, Mazziotta JC, Fox PT. Bias between MNI and Talairach coordinates analyzed using the ICBM-152 brain template. Hum Brain Mapp 2007;28(11):1194-205. doi: 10.1002/hbm.20345.
2. Alkan Y, Biswal BB, Alvarez TL. Differentiation between vergence and saccadic functional activity within the human frontal eye fields and midbrain revealed through fMRI. PLoS One 2011;6:e25866.
