# Supplementary material for: Exposure to different light wavelengths impacts cortical areas associated with oculomotor and attentional control
Source: PLoS One. 2026 Sep 15;21(9):e0358046. doi: 10.1371/journal.pone.0358046 (PMC13577468; doi:10.1371/journal.pone.0358046)
Supplement: Supplementary file 2 [file pone.0358046.s002.docx]

**Table S2.** Leave-one-subject-out analysis for the fALFF and ReHo intersection maps. The original values for the fALFF, based on all subjects, were F = 11.02, p < 0.01, and fot the ReHo intersection maps, were F = 31.62, p < 0.0001.

| **Results for the fALFF intersection maps** | | |
| --- | --- | --- |
| **left_out_subject** | **F_procedure** | **p_procedure** |
| 1 | 10.814747 | 0.004041 |
| 2 | 9.441309 | 0.006167 |
| 3 | 7.391741 | 0.012614 |
| 4 | 14.106291 | 0.001683 |
| 5 | 9.268653 | 0.006523 |
| 6 | 8.800344 | 0.007622 |
| 7 | 9.863894 | 0.004307 |
| **Results for the ReHo intersection maps** | | |
| **left_out_subject** | **F_procedure** | **p_procedure** |
| 1 | 23.819224 | 0.000254 |
| 2 | 50.004869 | 0.000013 |
| 3 | 25.835654 | 0.000186 |
| 4 | 22.249482 | 0.000329 |
| 5 | 35.156448 | 0.000056 |
| 6 | 23.766038 | 0.000256 |
| 7 | 26.85425 | 0.000095 |
