# Supplementary material for: Exposure to different light wavelengths impacts cortical areas associated with oculomotor and attentional control
Source: PLoS One. 2026 Sep 15;21(9):e0358046. doi: 10.1371/journal.pone.0358046 (PMC13577468; doi:10.1371/journal.pone.0358046)
Supplement: Supplementary file 3 [file pone.0358046.s003.docx]

**Table S3.** Regions of interest defined to map the area surrounding seed d (MNI −34, 7, 36). Only seeds 1 and 2 showed significant effects of light on fALFF (p < 0.05).

| **MNI** | **x** | **y** | **z** | **Area** |
| --- | --- | --- | --- | --- |
| seed 1* | -37 | 0 | 36 | Left_Premotor |
| seed 2* | -40 | 5 | 36 | Left_Premotor |
| seed 3 | -44 | 8 | 36 | Left_FEF |
| seed 4 | -44 | 2 | 40 | Left_Premotor |
| seed 5 | -50 | 8 | 43 | Left_Premotor |
| seed 6 | -50 | 2 | 41 | Left_Premotor |
| seed 7 | -39 | 3 | 36 | Left_Premotor |
