# Supplementary material for: Exposure to different light wavelengths impacts cortical areas associated with oculomotor and attentional control
Source: PLoS One. 2026 Sep 15;21(9):e0358046. doi: 10.1371/journal.pone.0358046 (PMC13577468; doi:10.1371/journal.pone.0358046)
Supplement: Supplementary file 4 [file pone.0358046.s004.docx]

**Table S4.** MNI coordinates adapted from Vernet et al. (2011) and Yeo et al. (2011) for FEF mapping (see Figure 2D).

| **X** | **Y** | **Z** | **Module** | **Source** |
| --- | --- | --- | --- | --- |
| -32 | -5 | 50 | 1 | Paus 1996,^29^ |
| -35 | -21 | 50 | 2 | Petit and Haxby, 1999,^28^ |
| -30 | 10 | 53 | 3 | Luna et al., 1998^,30^ |
| -38 | 26 | 30 | 4 | Tehovnik et al., 2000^,31^ |
| -42 | 12 | 36 | 5 | Ioannides et al., 2004^,32^ |
| -50 | 6 | 30 | 7 | PrCv from Yeo et al., 2011,^27^ |
| -26 | -6 | 48 | 7 | FEF from Yeo et al., 2011,^27^ |

**References**

1. Vernet M, Quentin R, Chanes L, Mitsumasu A, Valero-Cabré A. Frontal eye field, where art thou? Anatomy, function, and non-invasive manipulation of frontal regions involved in eye movements and associated cognitive operations. Front Integr Neurosci. 2014 Aug 22;8:66. doi: 10.3389/fnint.2014.00066. eCollection 2014.PMID: 25202241
2. Yeo BT, Krienen FM, Sepulcre J, Sabuncu MR, Lashkari D, Hollinshead M, Roffman JL, Smoller JW, Zöllei L, Polimeni JR, Fischl B, Liu H, Buckner RL. The organization of the human cerebral cortex estimated by intrinsic functional connectivity. J Neurophysiol 2011;106(3):1125-65. doi: 10.1152/jn.00338.2011. Epub 2011 Jun 8.PMID: 21653723.
