# Supplementary material for: Exposure to different light wavelengths impacts cortical areas associated with oculomotor and attentional control
Source: PLoS One. 2026 Sep 15;21(9):e0358046. doi: 10.1371/journal.pone.0358046 (PMC13577468; doi:10.1371/journal.pone.0358046)
Supplement: Supplementary file 5 [file pone.0358046.s005.docx]

**Table S5.** MNI coordinates for network mapping adapted from Yeo et al. (2011; see Figure 2E). The set includes all 24 seeds reported in Table 5 of Yeo et al. (2011), along with six additional seeds, primarily from the dorsal attention network (PrCv, FEF, aMT, SPLA, IPS2, IPS3m), derived from Table 2 of the same study. *p = 0.001.

| **X** | **Y** | **Z** | **Module** | **Size** | **Source** |
| --- | --- | --- | --- | --- | --- |
| -22 | -8 | 54 | 1 | 3 | DA_A |
| -34 | -38 | 44 | 1 | 3 | ***DA_B** |
| -18 | -69 | 51 | 1 | 3 | DA_C |
| -51 | -64 | -2 | 1 | 3 | DA_D |
| -8 | -63 | 57 | 1 | 3 | DA_E |
| -49 | 3 | 34 | 1 | 3 | DA_F |
| -50 | 6 | 30 | 1 | 3 | PrCv |
| -26 | -6 | 48 | 1 | 3 | FEF |
| -51 | -64 | 2 | 1 | 3 | aMT |
| -28 | -61 | 60 | 1 | 3 | SPLA |
| -40 | -37 | 42 | 1 | 3 | IPS2 |
| -31 | -48 | 46 | 1 | 3 | IPS3m |
| -31 | 39 | 30 | 2 | 3 | SAL_A |
| -54 | -36 | 27 | 2 | 3 | SAL_B |
| -60 | -59 | 11 | 2 | 3 | SAL_C |
| -5 | 15 | 32 | 2 | 3 | SAL_D |
| -8 | -24 | 39 | 2 | 3 | SAL_E |
| -31 | 11 | 8 | 2 | 3 | SAL_F |
| -40 | 50 | 7 | 3 | 3 | FP_A |
| -43 | -50 | 46 | 3 | 3 | FP_B |
| -57 | -54 | -9 | 3 | 3 | FP_C |
| -5 | 22 | 47 | 3 | 3 | FP_D |
| -6 | 4 | 29 | 3 | 3 | FP_E |
| -4 | -76 | 45 | 3 | 3 | FP_F |
| -27 | 23 | 48 | 4 | 3 | DM_A |
| -41 | -60 | 29 | 4 | 3 | DM_B |
| -64 | -20 | -9 | 4 | 3 | DM_C |
| -7 | 49 | 18 | 4 | 3 | DM_D |
| -25 | -32 | -18 | 4 | 3 | DM_E |
| -7 | -52 | 26 | 4 | 3 | DM_F |
