# Supplementary material for: Exposure to different light wavelengths impacts cortical areas associated with oculomotor and attentional control
Source: PLoS One. 2026 Sep 15;21(9):e0358046. doi: 10.1371/journal.pone.0358046 (PMC13577468; doi:10.1371/journal.pone.0358046)
Supplement: Supplementary file 6 [file pone.0358046.s006.docx]

**Figure S1**. ROIs surrounding the target (MNI, -34,7,36).
