# Supplementary material for: Exposure to different light wavelengths impacts cortical areas associated with oculomotor and attentional control
Source: PLoS One. 2026 Sep 15;21(9):e0358046. doi: 10.1371/journal.pone.0358046 (PMC13577468; doi:10.1371/journal.pone.0358046)
Supplement: Supplementary file 7 [file pone.0358046.s007.docx]

**Figure S2.** Intersection between fALFF and ReHo over Yeo’s Percellation
